# Why do burying beetles bury? Competition and the fitness benefits of parental care

**DOI:** 10.1093/beheco/arag100

**Published:** 2026-08-29

**Authors:** Erin Bolger, Paul R Martin

**Affiliations:** Department of Biology, Queen's University, 116 Barrie Street, Kingston, Ontario K7L 3N6, Canada; Department of Biology, University of Saskatchewan, 112 Science Place, Saskatoon, Saskatchewan S7N 5E2, Canada; Department of Biology, Queen's University, 116 Barrie Street, Kingston, Ontario K7L 3N6, Canada

**Keywords:** ecology, evolution, competition, parental care, species interactions

## Abstract

Parental care increases the fitness of current offspring at a cost to their parents, compromising parental resources for future reproduction. Given this inherent conflict and cost, complex parental care is relatively uncommon. Reliance on a rare and critical resource for reproduction that necessitates intense competition and defence could be a key reason for the evolution of costly and diverse parental care behaviors. Burying beetles (genus *Nicrophorus*) rely on carrion for reproduction, and have a suite of parental care behaviors, including the burying of small carcasses within underground breeding chambers. Carcass burial is energetically costly, but may confer fitness benefits to beetles by creating a safe, controlled environment for rearing offspring and securing the carrion. Yet, no study has directly tested whether carcass burial is adaptive, and if so, the selective pressures favoring this trait. Here, we manipulated the ability of beetle parents to bury carrion in nature to see the effect on reproductive success. Preventing beetles from burying led to complete reproductive failure, primarily due to competition with other invertebrates and vertebrates. Results align with previous ideas that competition played an important role in the evolution of carrion burial in *Nicrophorus* burying beetles, but do not exclude a role of other selective pressures (eg, microbial competition, microclimate). Competition is likely an important selective pressure driving the evolution of parental care, especially for species where intense competition for rare and critical resources persists throughout the growth and development of offspring, impacting the likelihood of reproductive failure.

## Teaser

Why do parents care for young when they could use that time and energy to produce more offspring? We addressed this question in burying beetles, examining parental burial of carrion that provides an essential resource for offspring. When we prevented beetles from burying, they reared no offspring due to competition with other species for carrion. Our results suggest competition can favor the evolution of parental care when prolonged resource defence is essential for offspring survival.

## Introduction

Parental care increases the fitness of current offspring at a cost to their parents' survival or future reproductive success. Parents tending to offspring can be at a greater risk of depredation, have less energy to devote to themselves, and have reduced time and resources for other reproductive efforts (Shaffer and Formanowicz 1996; Li and Jackson 2003; Alonso-Alvarez and Velando 2012; López-Idiáquez et al. 2018). These costs to parents are likely what make parental care rare in animals, with most animals having no parental care and instead investing in multiple broods to maximize reproductive success (Clutton-Brock and Scott 1991). Nonetheless, parental care has evolved multiple times and is widespread and complex in some animal groups (Balshine 2012; Trumbo 2012). The repeated evolution and persistence of parental care across diverse lineages suggests that the same conditions might repeatedly and independently promote its evolution. Indeed, ecological factors that threaten offspring survival or limit their growth, such as harsh abiotic conditions, high predation, and rare and limiting resources, are thought to be key drivers of the evolution of parental care (Klug and Bonsall 2010).

Competition is often neglected in studies of the causes of parental care, yet could drive its evolution through the effect of competitive interactions on reproductive success (Keddy 2012). Competition is ubiquitous in nature—all organisms depend on limited resources, such as food or nesting sites (Keddy 2012), and parents must successfully compete for those resources in order to reproduce. If competition persists throughout the duration of a vulnerable offspring stage, then parental care might evolve or increase to ensure offspring growth and survival (Klug and Bonsall 2014). For example, birds that use rare, preexisting cavities for nesting must initially compete for cavities, and then continue to defend them against individuals that will usurp their nest site at any stage of reproduction (Martin 1997; Olsen et al. 2008; Cockle et al. 2010; Gerstmayer et al. 2022). Similarly, parents of predatory species whose offspring are poor hunters and ill-equipped to compete for food can improve offspring success by providing them with food throughout development (Gardner and Smiseth 2011). Organisms that depend on rare, ephemeral, or limiting resources can also face intense competition to secure the resource initially, and continuing while offspring use the resource to grow and develop, favoring parents that remain with their offspring and provide care (Royle et al. 2012). The outcome of competition for key resources before and during offspring growth and development can thus determine reproductive success, driving the evolution of parental care.

The *Nicrophorus* burying beetles (Coleoptera: Staphylinidae: Silphinae) are a genus where competition for limited resources is intense and persists throughout larval development, and where the outcome of competitive interactions can determine reproductive success. *Nicrophorus* burying beetles depend on small, vertebrate carcasses to reproduce (Pukowski 1933; Potticary et al. 2024). Beetles must first locate a carcass and defend it against other competitors, such as vertebrate, invertebrate, and microbial scavengers, that also use carcasses for food or reproduction. Carrion is rare, leading to intense competition within and among species (Trumbo 1990). *Nicrophorus* beetles engage in parental care behaviors such as rolling the carcass into a ball, defurring the carcass, coating it in anal and oral antimicrobial secretions, and burying it underground (Potticary et al. 2024). The female then lays eggs in the soil nearby and the pair feeds their larvae regurgitated carrion before young can feed on the carrion themselves.

A central component of the *Nicrophorus* parental care strategy is the burial of the carcass. Burial itself is not necessary for reproductive success: *Nicrophorus* beetles regularly breed successfully in the laboratory without carcass burial, where they are protected from most environmental selective pressures (Trumbo 2016a). Nonetheless, burial is thought to provide fitness benefits by reducing competition and predation from other invertebrates and vertebrates (Pukowski 1933; Trumbo and Sikes 2021). Burial could also reduce microbial competition; unburied piglet carrion had a faster initial rate of decay compared with buried carrion (Miles 2019), and cooler underground temperatures could slow the activity of microbial competitors (Lomander et al. 1998). Other potential benefits to burial include providing a suitable microclimate for the development of larvae (eg, protecting against high or variable temperatures; Quinby et al. 2020; Harrison 2021; Keller et al. 2021) and providing a structural chamber that could facilitate adult attendance and maintenance of the carcass and offspring. Overall, some evidence supports all of these ideas for why the carcass burial is advantageous for burying beetles, but the fitness benefits of carcass burial by beetles have never been directly tested in nature.

Here, we conducted field experiments to test the fitness consequences of burying in *Nicrophorus orbicollis*, a species of burying beetle that shows obligate biparental care and pronounced burying behavior (Potticary et al. 2024). We first tested the hypothesis that carcass burial is adaptive (H_1_), as opposed to providing no fitness benefits (H_0_) or being maladaptive (H_2_, eg, if the trait was adaptive historically, but is no longer); the latter 2 results would challenge the overwhelming view of parental care as an adaptive trait across species. We found support for H_1_; carcass burial was adaptive, and beetles prevented from burying were never able to successfully raise offspring. We also found that invertebrate and vertebrate scavenging caused the failure in the nonburial treatment, supporting previous ideas that a primary function of burying the carcass is to reduce competition from invertebrates and vertebrates.

## Methods

### Study species

*Nicrophorus orbicollis* shows obligate biparental care and pronounced burying behavior, providing an extreme example of parental care behaviors and carcass burial among *Nicrophorus* beetles (Capodeanu-Nägler et al. 2016; Potticary et al. 2024). This species is abundant at our study site (Burke et al. 2020) and reproduces from mid-May until late August (Wettlaufer et al. 2021). Competition is important for *Nicrophorus* species generally due to their reliance on carrion (Potticary et al. 2024), and among burying beetles, *N. orbicollis* have strong competitive abilities (Trumbo and Bloch 2002; Schrempf et al. 2021; Vangenne et al. 2023). Other *Nicrophorus* species at our site avoid competition for carcasses by using challenging habitats or times of year, whereas *N. orbicollis* frequently excludes these other species from carcasses in optimal habitats and seasons, where it has a competitive advantage due to its larger size and greater abundance (Wettlaufer et al. 2023; Burke et al. 2024).

### Study site

We conducted field experiments on the Queen's University Biological Station (QUBS; 44.5653, −76.3242, 115–170 m elevation) properties near Elgin, Ontario, Canada. For the first experiment, we used a paired design with approximately 80 m between paired control and treatment (nonburial) sites, and at least 400 m separating each treatment pair from others for independence. We selected sites in mesic forest habitats with an abundance of leaf litter and avoided sites at the top of hills and open, grassy areas, where *N. orbicollis* is less common (Burke et al. 2020).

### Field methods: fitness benefits of burial

We used reproductive success as an estimate of relative fitness and compared the reproductive success of beetle parents prevented from burying carcasses (nonburial treatment) with those allowed to bury (control). We constructed a breeding apparatus using segments of 15.24 cm diameter polyvinyl chloride (PVC) pipe long enough for *N. orbicollis* to bury a carcass without vertical constraint (40 cm deep, Burke et al. 2024) (Fig. S1a). We blocked one end of the tube with wire mesh, allowing the beetles access to 2 cm of soil, which was inadequate to bury a mouse carcass, but adequate for laying eggs in the soil. In the nonburial treatment, the tube was oriented with the mesh at the top, preventing complete burial of the carcass, and in the control treatment, the tube was oriented with the mesh at the bottom, allowing carcass burial.

We completely buried the tubes in the ground (Fig. S1), and placed mouse (*Mus musculus*) carcasses (15–30 g) on the soil surface of each tube. We briefly attached a flowerpot of the same diameter as the tube, with the bottom removed, to the top of each tube using duct tape to prevent the beetles from removing the mouse from the tube (Fig. S1b, c). We used 2 different approaches to obtain beetle pairs for experiments. In the first year (2023), we allowed beetles to arrive at the sites on their own, which replicates natural conditions of arrival and mate choice, but has the potential to lead to variation in reproductive success among treatments attributable to differences in beetle pairs (eg, if only poor-quality pairs settle for a carcass that they cannot bury). In the second year (2024), we instead placed a randomly selected male and female beetle at each site, which removed differences in beetle pairs across treatments, but also the natural patterns of arrival and mate choice. The use of both methods provided a balance of natural conditions and controlled design, increasing our confidence in the results.

We checked the mice each day for evidence that *N. orbicollis* pairs had started reproducing, and we used behaviors such as defurring, balling up the carcass, and movement of the carcass as evidence for the initiation of breeding. We removed the flowerpot 3 d after the initiation of breeding to ensure the beetles were committed to breeding at that location and would not move the carcass (Fig. S1d). We returned 10 d following the initiation of breeding to collect third instar larvae for counting and weighing. We estimated relative fitness for each beetle pair using 2 measures of reproductive success: the number of offspring and average larval mass (g). If burial is adaptive (H_1_), then beetles prevented from burying should have reduced fitness; other hypotheses predict the opposite pattern (H_2_, trait is maladaptive) or no difference among treatments (H_0_).

We recorded scavenger presence, carcass desiccation, and reproductive behavior of the beetles at each site to provide information on the selective pressures differentially impacting beetle parents. We inferred vertebrate presence when the mouse was removed from a site and there was evidence of digging or disturbance. We assessed invertebrate presence during our daily site checks, and recorded the species, number, and activity of any invertebrates present. We also recorded the status of the carcass (eg, observations of carcass consumption, desiccation, and removal). Our sample sizes for each year and treatment are: burial treatment (*N* = 42 total; 2023, *N* = 30; 2024, *N* = 12); nonburial treatment (*N* = 40 total; 2023, *N* = 28; 2024, *N* = 12).

### Field methods: vertebrate exclusion

We tested the importance of vertebrate competition to the fitness benefits of burial by excluding vertebrates from an additional nonburial treatment in 2024 (we also attempted to exclude invertebrate competitors and prevent desiccation, but these treatments were unsuccessful; see Supplemental Material). We constructed a cage out of chicken wire that surrounded a nonburial site and secured it to the ground using tent stakes, preventing vertebrates from accessing the carcass and removing it from the tube. We followed the same procedure as described above to test the fitness of beetles prevented from burying when also protected from vertebrates. Our sample sizes are: nonburial, no vertebrate exclusion treatment (*N* = 12); nonburial, vertebrate exclusion treatment (*N* = 12).

### Statistical methods

We conducted statistical analyses in R (version 4.4.1, R Core Team 2024). To test the differences in outcome between treatments, we initially used binomial generalized linear mixed-effects models (glmer) (function “glmer” in the R package lme4, version 1.1-36; Bates et al. 2015). We tested 3 binomial response variables: reproductive success (“success”, where 1 = successfully raised third instar offspring and 0 = failed to raise third instar offspring), failure due to vertebrate competition, and failure due to invertebrate competition (“vertebrate” and “invertebrate” respectively, where 1 = failure due to vertebrate/invertebrate competition and 0 = otherwise). The predictors for each model were treatment (burial or nonburial), year, and their interaction (as fixed effects), and site (ie, a site with a burial and nonburial treatment pair) as a random effect to control for differences among sites. We included year as a fixed effect rather than a random effect because we had data for only 2 yr (ie, 2 levels; Bolker 2008) (see Supplemental Materials for an alternative approach that analyzed years separately, which yielded qualitatively similar results). In the vertebrate and invertebrate models, we also added average temperature in an interaction with treatment (treatment × temperature; see Supplemental Methods) as a fixed effect in the global model because temperature can affect the relative scavenging between vertebrates and invertebrates (DeVault and Rhodes 2002; DeVault et al. 2004, 2011). We did not consider the interaction between year and temperature because the temperatures in our experiment did not vary much between years; we did not consider the 3-way interaction for this same reason, and because of our limited sample size. In most cases, a treatment had no variance (eg, when all nonburial treatments failed), models did not converge, or models showed problems with fit, so we switched to Firth's Bias-Reduced Logistic Regressions (logistf) (function “logistf” from the R package logistf, version 1.26.0; Heinze et al. 2023) and estimated the significance of predictors using the “anova” function in the logistf package. To test for treatment × temperature effects, we used the “anova” function to compare models with and without treatment × temperature (all models included treatment × year; see Supplemental Methods). To test for year, treatment, and treatment × year effects, we used the “anova” function to compare models with treatment × year to models without each predictor. We used the penalized likelihood ratio (PLR) method (following Heinze et al. 2023) because our different model subsets had different likelihood ratio scales and thus were not directly comparable. We used the function “emmeans” in the R package emmeans (version 1.10.7; Lenth 2024) to estimate marginal means and *P*-values across levels (eg, treatment effects in different years), using a Tukey's Honestly Significant Difference (HSD) post-hoc test for each contrast. See Supplemental Methods for assessments of model fit.

Vertebrate scavengers removed carcasses completely, which prevented invertebrates from accessing the carcass afterwards. To control for the effect of vertebrates on invertebrates, we removed the sites with outcomes of failure due to vertebrates from the dataset and reran the model testing the impact of burial on breeding failure due to invertebrate competition using the same approaches described for tests above. We did not include temperature in these models, because we were only interested in the impact of vertebrates on invertebrates. We tested if experimentally excluding vertebrates increased failure due to invertebrates (2024 only) using a binomial generalized linear model (glm) with failure due to invertebrates (0 = no, 1 = yes) as the response variable, and treatment (nonburial, no vertebrate exclusion [NB] vs nonburial, vertebrate exclusion [NBV]) as the predictor.

## Results

### Fitness benefits of burial

#### Reproductive success

Beetle pairs allowed to bury their carcass had higher reproductive success than beetle pairs that were not allowed to bury (Firth's Bias-Reduced Logistic Regression (logistf), overall treatment effect, χ^2^ = 38.20, df = 2, *P* < 0.001, Fig. 1). None of the beetle pairs in the nonburial treatment successfully reared young to third instar (*N* = 40), compared with 59.5% success for the burial treatment (*N* = 42) (Fig. 1). We found no evidence for variation in success across years (logistf, overall year effect, χ^2^ = 0, df = 2, *P* = 1.0), or variation in treatment effects across years (treatment × year effect, χ^2^ = 0, df = 1, *P* = 1.0; 2023: burial, 60% of beetle pairs succeeded [*N* = 30]; nonburial, 0% of pairs succeeded [*N* = 28]; 2024: burial, 58% of pairs succeeded [*N* = 12]; nonburial, 0% of pairs succeeded [*N* = 12]). See Supplemental Results for the results of models separated by year, which yielded qualitatively similar results, with strong treatment effects in each year.

**Figure 1:**
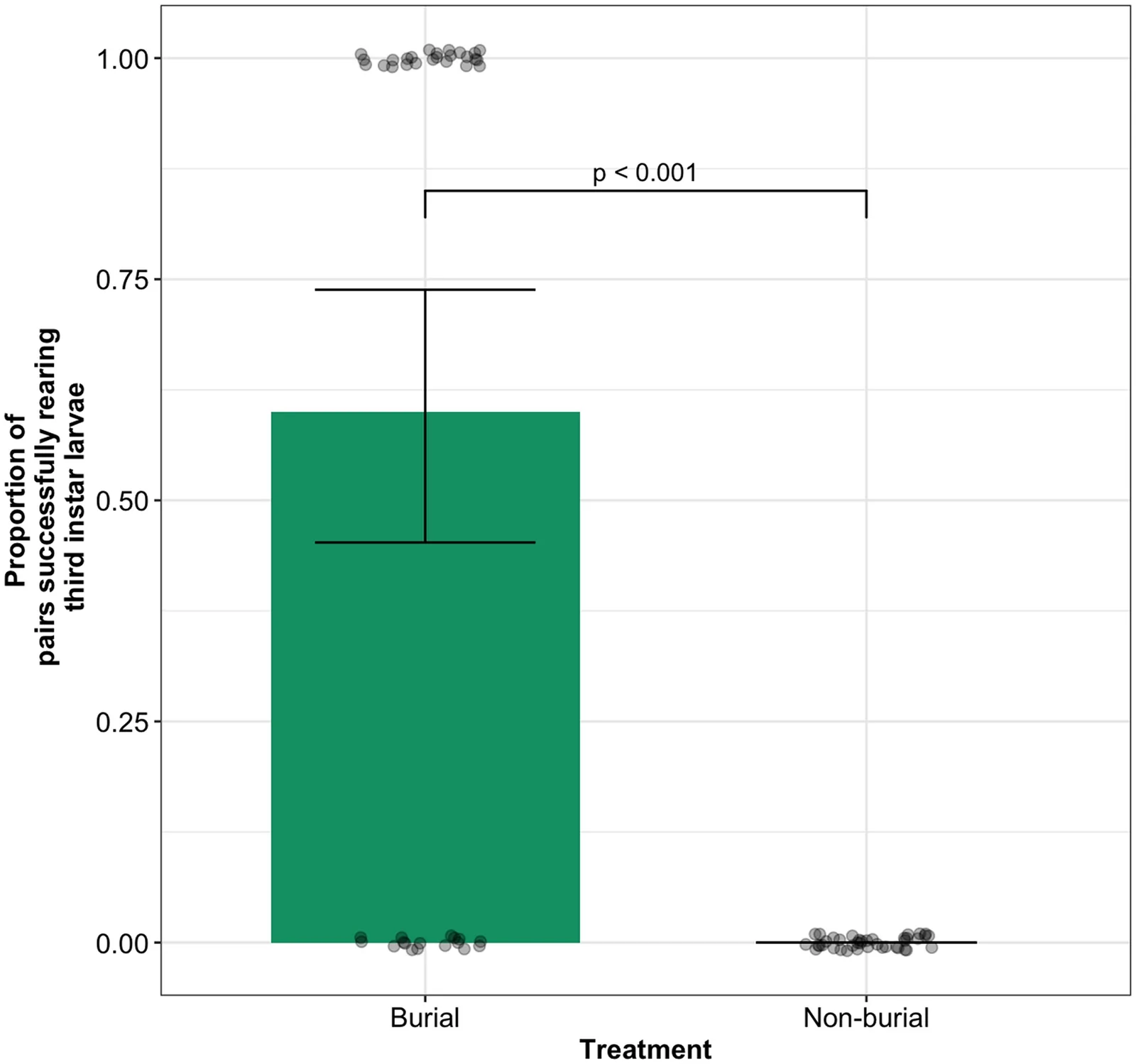
*Nicrophorus orbicollis* pairs that could bury their carcasses had higher reproductive success than pairs prevented from burying. Bars represent the proportion of breeding pairs that were able to successfully rear third instar larvae for burial (*N* = 42 pairs) and nonburial treatments (*N* = 40 pairs), combined from 2023 and 2024. Error bars represent 95% confidence intervals. Points represent raw data points (1 = successful pair, 0 = unsuccessful pair).

#### Failure due to vertebrate competition

Beetle pairs allowed to bury their carcasses were less likely to have their carcass scavenged by vertebrates (logistf, overall treatment effect: χ^2^ = 6.56, df = 2, *P* = 0.038), with the effects of vertebrate competition varying across years (logistf, overall year effect, χ^2^ = 7.09, df = 2, *P* = 0.029), and weak evidence for variation in treatment effects across years (logistf, treatment × year effect, χ^2^ = 1.86, *df* = 1, *P* = 0.17; Fig. 2a, b; estimated marginal means (probability of failure due to vertebrate competition) and treatment contrasts from logistf, 2023: burial = 0.145, SE = 0.063, nonburial = 0.224, SE = 0.077; contrast, odds.ratio = 0.588, SE = 0.398, *t.ratio* = −0.79, *P* = 0.43; 2024: burial = 0.192, SE = 0.109, nonburial = 0.654, SE = 0.132; contrast, odds.ratio =0.126, SE = 0.115, *t.ratio* =−2.27, *P* = 0.026). Overall, 35.0% of beetle pairs that were prevented from burying their carcasses failed due to vertebrate scavenging, compared with only 14.3% of beetle pairs that were allowed to bury. Separating models by year revealed strong evidence for higher vertebrate scavenging of unburied vs buried carcasses in 2024, and weak evidence in 2023 (Supplemental Results).

**Figure 2:**
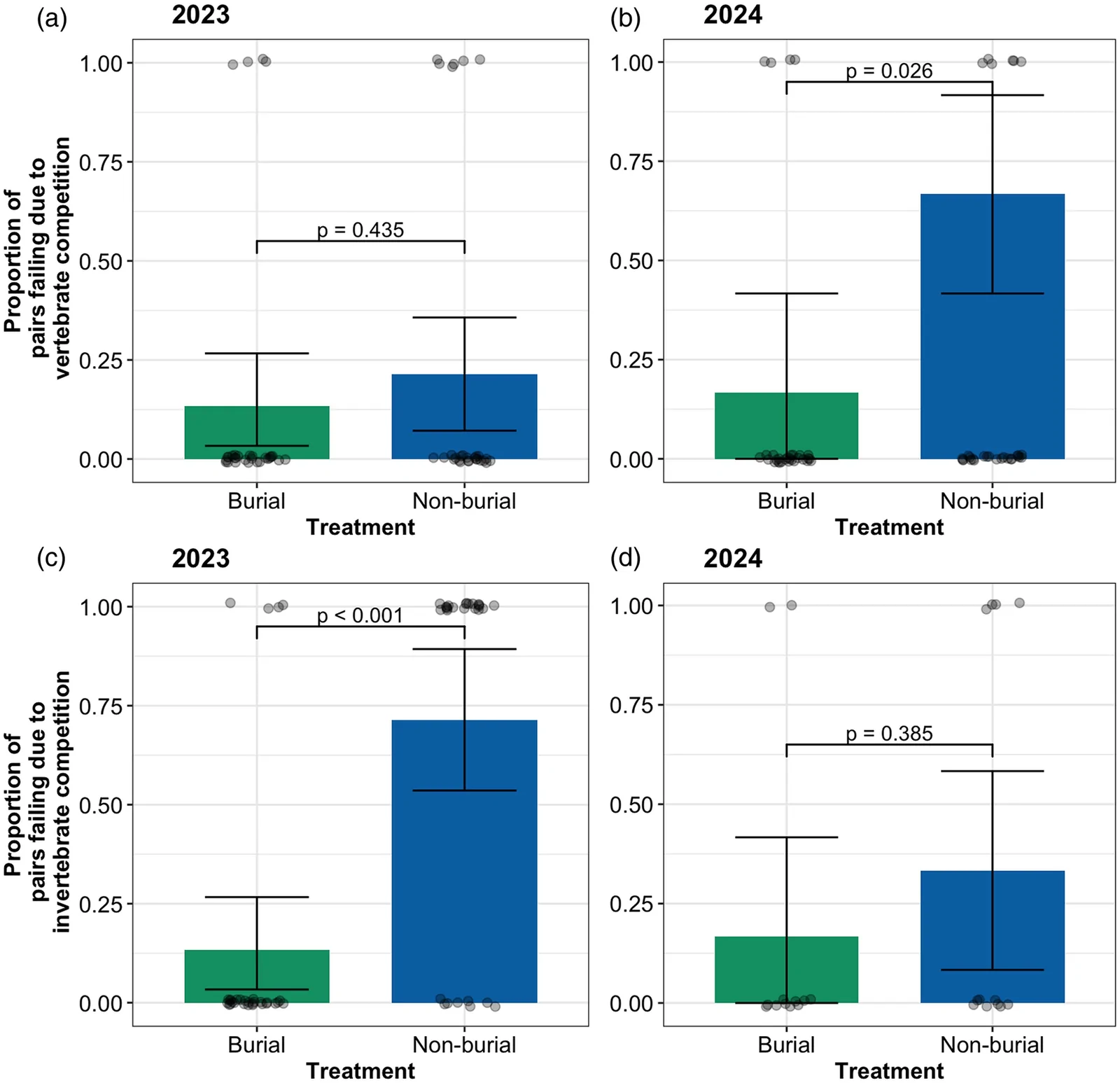
*Nicrophorus orbicollis* pairs that could bury their carcasses had fewer instances of vertebrates (a, b) and invertebrates (c, d) causing reproductive failure than pairs prevented from burying. Bars represent the proportion of pairs that failed due to a) vertebrate competition in 2023 for the burial treatment (*N* = 30 pairs) and nonburial treatment (*N* = 28 pairs), b) vertebrate competition in 2024 for the burial treatment (*N* = 12 pairs) and nonburial treatment (*N* = 12 pairs), c) invertebrate competition in 2023 for the burial treatment (*N* = 30 pairs) and nonburial treatment (*N* = 28 pairs), and d) invertebrate competition in 2024 for the burial treatment (*N* = 12 pairs) and the nonburial treatment (*N* = 12 pairs). Error bars represent 95% confidence intervals. *P*-values are from treatment × year contrasts (emmeans) on Firth's Bias-Reduced Logistic Regression models (see Results). Points represent raw data points (1 = pair failed due to vertebrate (a, b) or invertebrate (c, d) competition, 0 = pair did not fail due to vertebrate (a, b) or invertebrate (c, d) competition).

#### Failure due to invertebrate competition

Beetle pairs allowed to bury were less likely to fail due to invertebrate competition (logistf, overall treatment effect, χ^2^ = 21.39, df = 2, *P* < 0.001). We found weak evidence for variation in invertebrate competition across years (logistf, overall year effect, χ^2^ = 4.44, df = 2, *P* = 0.11) and for treatment effects varying across years (logistf, treatment × year effect, χ^2^ = 2.02, df = 1, *P* = 0.16; Fig. 2c, d; estimated marginal means (probability of failure due to invertebrate competition) and treatment contrasts from logistf, 2023: burial = 0.145, SE = 0.063, nonburial = 0.707, SE = 0.085; contrast, odds.ratio = 0.070, SE = 0.046, *t.ratio* = −4.06, *P* < 0.001; 2024: burial = 0.192, SE = 0.109, nonburial = 0.346, SE = 0.132; contrast, odds.ratio = 0.450, SE = 0.411, *t.ratio* = −0.87, *P* = 0.38). Overall, 60.0% of beetle pairs that were prevented from burying their carcasses failed due to invertebrate scavenging, compared with only 14.3% of beetle pairs that were allowed to bury. Separating models by year revealed qualitatively similar results, with strong evidence for treatment effects in 2023, and weak evidence in 2024 (Supplementary Results). Carcasses with beetle pairs that failed due to invertebrate competition were also frequently desiccated, though desiccation never occurred on its own (ie, without invertebrate presence) to cause reproductive failure for a beetle pair.

#### Effect of temperature

Temperature did not predict the likelihood of failure due to vertebrate or invertebrate competition (logistf, temperature × treatment effect; vertebrate competition: χ^2^ = 0.34, df = 2, *P* = 0.84; invertebrate competition: χ^2^ = 0.50, df = 2, *P* = 0.78).

#### Effect of vertebrates on invertebrates

Vertebrate activity affected the likelihood of failure due to invertebrate competition. After removing pairs that failed due to vertebrates, beetle pairs allowed to bury were less likely to fail due to invertebrate competition, with no year effect (logistf, overall treatment effect, χ^2^ = 36.04, df = 2, *P* < 0.001; treatment × year effect, χ^2^ = 0, df = 1, *P* = 1.0; year effect χ^2^ = 0, df = 2, *P* = 1.0, Fig. 3a; estimated marginal means (probability of failure due to invertebrate competition) and treatment contrasts from logistf, 2023: burial = 0.167, SE = 0.072, nonburial = 0.891, SE = 0.065; contrast, odds.ratio = 0.024, SE = 0.021, t.ratio = −4.39, *P* < 0.001; 2024: burial = 0.227, SE = 0.126, nonburial = 0.900, SE = 0.134; contrast, odds.ratio = 0.033, SE = 0.054, t.ratio = −2.07, *P* = 0.043). Vertebrate exclusion experiments further supported an impact of vertebrates on invertebrate competition, with beetle pairs failing more often due to invertebrate competition once vertebrate competitors were excluded, with 92% of beetle pairs in the nonburial treatment with vertebrate exclusion failing due to invertebrate competition (*N* = 12), compared with 33% of pairs in the nonburial treatment without vertebrate exclusion (*N* = 12) (binomial glm: *z* = 2.55, *P* = 0.01, Fig. 3b).

**Figure 3:**
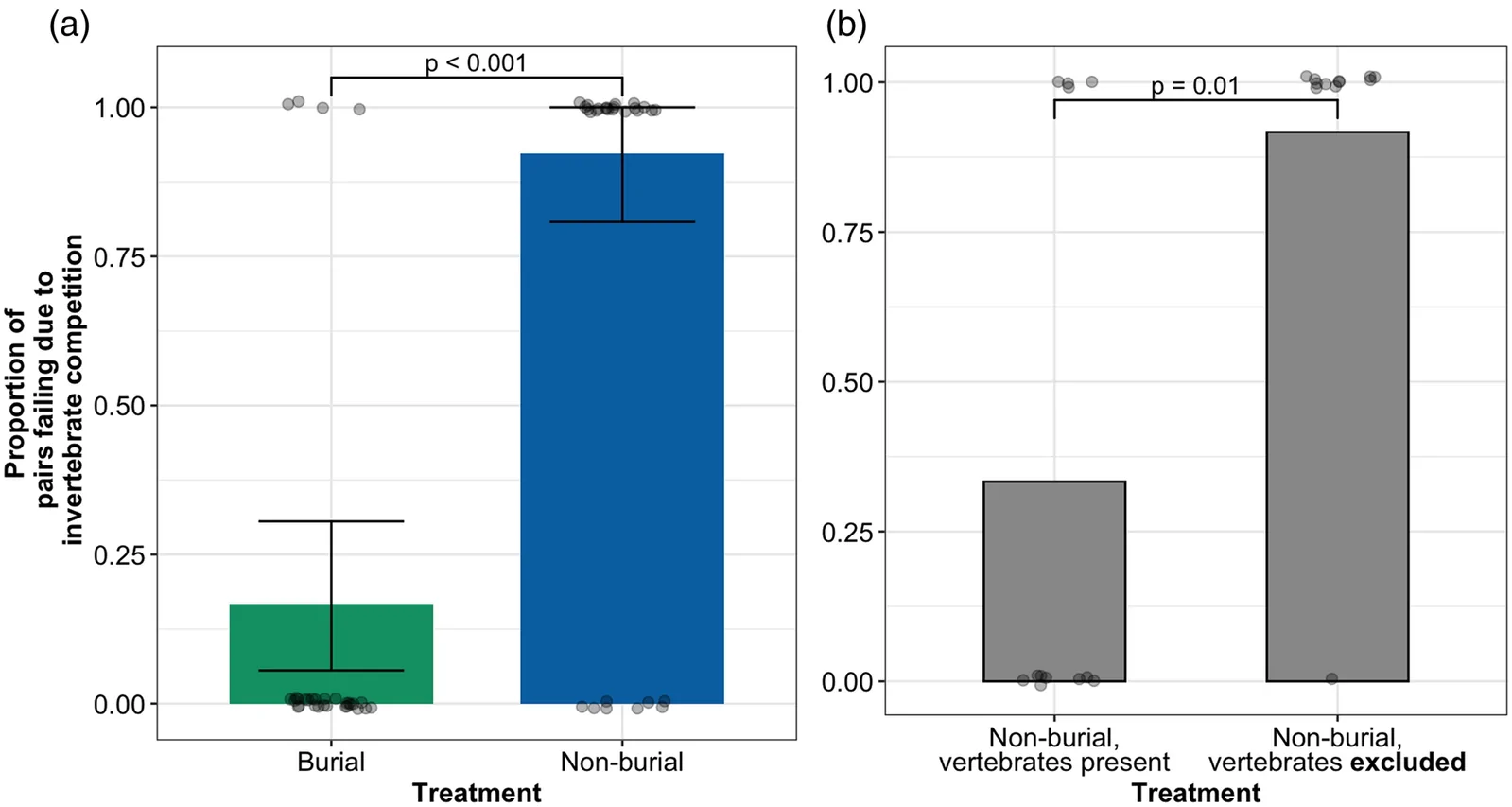
Vertebrates influence invertebrate competition. a) After removing outcomes of pairs failing due to vertebrate competition, *Nicrophorus orbicollis* pairs that could bury their carcasses had fewer instances of invertebrates causing reproductive failure than pairs prevented from burying, with no year difference. Bars represent the proportion of pairs that failed to rear third instar offspring due to invertebrate competition in burial (*N* = 36 pairs) and nonburial treatments (*N* = 26 pairs), combined from 2023 and 2024, after removing pairs that failed due to vertebrate scavengers. Error bars represent 95% confidence intervals. b) When vertebrates were experimentally excluded, more burying beetle pairs failed due to invertebrate competition, indicating a direct effect of vertebrate exclusion on invertebrate competition. Bars represent proportion of pairs that failed to rear third instar offspring due to invertebrate competition in nonburial (*N* = 12) and nonburial (vertebrates excluded) (*N* = 12) treatments.

## Discussion

We tested if burying carrion provides fitness benefits to *Nicrophorus orbicollis* in the field by manipulating the ability of beetle parents to bury carrion. We found that beetles prevented from burying had no reproductive success compared with 59.5% success for beetles allowed to bury. Beetle pairs that were prevented from burying had a 3.3 times higher failure rate due to invertebrate competition and a 1.5 times higher failure rate due to vertebrate competition, with invertebrate scavenging causing greater failure of unburied carcasses in 2023, and vertebrate scavenging causing greater failure of unburied carcasses in 2024. Our results suggest that burial of carcasses is an adaptation for reproduction in burying beetles, and is particularly important for reducing competition with invertebrate and vertebrate scavengers. Other factors (eg, reduced microbial competition, improved conditions for egg and larval development, and provision of a physical brood chamber) could provide secondary and important fitness benefits to burial; however, none of these are likely to have a larger effect on fitness than the complete reproductive failure that resulted from competition with invertebrates and vertebrates for unburied carcasses. Burial itself is not necessary for successful breeding in *N. orbicollis*; pairs regularly breed successfully without burial in captivity, where they are protected from environmental challenges (Trumbo 2016a).

### Invertebrate and vertebrate competition

Invertebrate competition was the main cause of reproductive failure for beetle pairs in the nonburial treatment, primarily through consumption of the carcass, and secondarily through hastening carcass desiccation by increasing exposed carcass surface area and reducing carcass mass. The main invertebrates that we observed competing with burying beetles were blow and flesh flies (Diptera: Calliphoridae, Sarcophagidae) and non*Nicrophorus* carrion beetles (Silphinae: *Necrophila*, *Oiceoptoma*), and we observed only small flies (likely phorid flies, Phoridae) with carcasses once *Nicrophorus* had buried the carcass, suggesting burial was effective at greatly reducing invertebrate competition. *Nicrophorus* species can prevent the colonization of their carcasses by eating the eggs and larvae of other invertebrate species already present on the carcass (Scott 1998) and by killing or dismembering potential invertebrate competitors and predators (Scott 1998; Suzuki 2000); however, when we prevented *Nicrophorus* from burying, other invertebrates accumulated in large numbers and appeared to overwhelm *Nicrophorus*’ ability to defend their carcasses. The process of burial and other preparation behaviors that accompany burial likely involve the removal of eggs or larvae of species that have already colonized the carcass (Potticary et al. 2024), and buried carrion is detected much less frequently by other silphids (Shubeck and Blank 1982; Shubeck 1985). Carrion-using invertebrates locate carrion by detecting emitted volatile compounds (Verheggen et al. 2017); thus, burial reduces subsequent (post-burial) invertebrate competition by reducing the spread of the compounds that attract invertebrate competitors to the carcass (Trumbo and Sikes 2021; Trumbo et al. 2021). Burial could also prevent any invertebrate that cannot dig through the soil from accessing the carcass.

Vertebrate scavengers are another primary competitor with burying beetles for carcasses, and are the main consumers of small vertebrate carcasses in some regions and at some times of year (DeVault and Rhodes 2002; DeVault et al. 2004; Trumbo 2016b). Vertebrates arrived at carcasses early and scavenged carcasses in their entirety, precluding burying beetles from using a carcass for reproduction (DeVault et al. 2003; Ponce et al. 2010; Turner et al. 2017). Vertebrate consumption of a carcass may also result in the depredation of adult burying beetles and their offspring (Trumbo and Bloch 2000). Vertebrates detect carcasses primarily through odor cues, and burial reduces the spread of volatile compounds emitted from the carcasses, thus limiting vertebrate detection of carcasses (Ki et al. 2018; Trumbo and Sikes 2021; Trumbo 2023); experiments involving carrion burial have reduced vertebrate scavenging (burial by wild cats: Bischoff-Mattson and Mattson 2009; Teurlings et al. 2020; experimental burial by humans: Trumbo and Sikes 2021). Burying is likely the most effective way of deterring vertebrate scavengers, as carcass preparation alone (excluding burial) does not appear to reduce vertebrate scavenging, despite modifying the odor profile of the carcass through carcass preparation (Trumbo and Sikes 2021; Trumbo et al. 2021). In our study, we found a smaller invertebrate effect on beetle failure in 2024 that was statistically explained by the higher vertebrate scavenging in that year, suggesting that vertebrates impact invertebrate use of carcasses generally. Experimentally excluding vertebrates from unburied carcasses increased failure rates for unburied carcasses due to invertebrate scavenging from 33% to 92% (Fig. 3b), supporting a direct impact of vertebrates on invertebrate carcass use.

### Competition and parental care

Parents that provide offspring with resources, such as food or nest sites, often face competition for those resources. Selection may then favor parents that monopolize and protect these resources from competitors, increasing parental care (Clutton-Brock and Scott 1991; Alonso-Alvarez and Velando 2012). To monopolize resources and defend against competitors, parents must remain close to the resource, and thus, their offspring, which could further promote the evolution of other parental care behaviors, such as food provisioning or defense of young (Choe and Crespi 1997; Gardner and Smiseth 2011). Increased parental attendance can also lead to offspring becoming more dependent on care, impeding the loss of parental care once it has evolved (Royle et al. 2012). Resource monopolization is thus a key component of the evolution of parental care—parental care is most common where species have monopolizable resources available to them, such as discrete or clumped resources that can be defended (Keenleyside 1991; Székely and Reynolds 1995; Gubernick 2013). When these resources are in high-demand, defending against competitors could become a primary component of parental care strategies.

Parental care may thus function as a trait to reduce the fitness costs of competition, and several previous studies have demonstrated an increase in parental care with increased competitive pressure. For example, birds increase offspring feeding or nest defense behaviors in response to increased competition for nest sites (Jacot et al. 2009; Goldshtein et al. 2018). Neotropical damselflies that used sites under high competitive pressure also provided parental care in the form of offspring defense, whereas damselflies that used less favorable sites provided no parental care (Fincke 1992). However, few studies directly link competition to parental care, and we know of no previous study to provide experimental evidence of fitness benefits to parental care as a result of reduced interspecific competition. Our results, along with previous work demonstrating increased parental care in response to competitive pressure, suggest that competition could be an important driver of the evolution of parental care.

Competition likely plays an important role in the evolution of parental care in species that use relatively benign or desirable resources and environments. In these environments, success and persistence depends primarily on resource defense, thus favoring the evolution of competitive ability at the expense of adaptations to challenge (Martin and Ghalambor 2023, 2024). Our results provide evidence that parental care can be an important trait contributing to competitive ability, particularly when intense competition persists throughout offspring development and when resources can be defended. Parental care could provide additional benefits if it improves the competitive ability of offspring in adulthood, such as by increasing their size at maturity (Klug and Bonsall 2014). When parental care functions to confer or improve competitive ability, we should expect it to be more prevalent and complex in competitively dominant species, allowing them to use and monopolize resources and environments preferred by many ecologically similar species.

## Supplementary Material

arag100_Supplementary_Data

## Data Availability

Analyses reported in this article can be reproduced using the data provided by Bolger and Martin (2026).

## References

[arag100-B1] Alonso-Alvarez C, Velando A. 2012. Benefits and costs of parental care. In: Royle NJ, Smiseth PT, editors. The evolution of parental care. Oxford University Press. p. 81–100.

[arag100-B2] Balshine S . 2012. Patterns of parental care in vertebrates. In: Royle NJ, Smiseth PT, editors. The evolution of parental care. Oxford University Press. p. 62–80.

[arag100-B3] Bates D, Mächler M, Bolker BBen, Walker S. 2015. Fitting Linear Mixed-Effects Models Using **lme4**. Journal of Statistical Software. 67. 10.18637/jss.v067.i01.

[arag100-B4] Bolger E, Martin PR. 2026. Data from: Why do burying beetles bury? Competition and the fitness benefits of parental care. Behavioural Ecology. 10.17605/OSF.IO/X7JUK.

[arag100-B5] Bischoff-Mattson Z, Mattson D. 2009. Effects of simulated mountain lion caching on decomposition of ungulate carcasses. West N Am Nat. 69:343–350. 10.3398/064.069.0308.

[arag100-B6] Bolker BM . 2008. Ecological models and data in R. Princeton University Press.

[arag100-B7] Burke KW, Groulx AF, Martin PR. 2024. The competitive exclusion–tolerance rule explains habitat partitioning among co-occurring species of burying beetles. Ecology. 105:e4208. 10.1002/ecy.4208. 37948189

[arag100-B8] Burke KW, Wettlaufer JD, Beresford DV, Martin PR. 2020. Habitat use of co-occurring burying beetles (genus *Nicrophorus*) in southeastern Ontario, Canada. Can J Zool. 98:591–602. 10.1139/cjz-2019-0287.

[arag100-B9] Capodeanu-Nägler A et al 2016. From facultative to obligatory parental care: interspecific variation in offspring dependency on post-hatching care in burying beetles. Sci Rep. 6:29323. 10.1038/srep29323. PMC4932505 27378180

[arag100-B10] Choe JC, Crespi BJ. 1997. The evolution of social behavior in insects and arachnids. Cambridge University Press.

[arag100-B11] Clutton-Brock TH, Scott D. 1991. The evolution of parental care. Princeton University Press.

[arag100-B12] Cockle KL, Martin K, Drever MC. 2010. Supply of tree-holes limits nest density of cavity-nesting birds in primary and logged subtropical Atlantic forest. Biol Conserv. 143:2851–2857. 10.1016/j.biocon.2010.08.002.

[arag100-B13] DeVault TL, Brisbin IL Jr, Rhodes OE Jr. 2004. Factors influencing the acquisition of rodent carrion by vertebrate scavengers and decomposers. Can J Zool. 82:502–509. 10.1139/z04-022.

[arag100-B14] DeVault TL, Olson ZH, Beasley JC, Rhodes OE. 2011. Mesopredators dominate competition for carrion in an agricultural landscape. Basic Appl Ecol. 12:268–274. 10.1016/j.baae.2011.02.008.

[arag100-B15] DeVault TL, Rhodes OE. 2002. Identification of vertebrate scavengers of small mammal carcasses in a forested landscape. Acta Theriol (Warsz). 47:185–192. 10.1007/BF03192458.

[arag100-B16] DeVault TL, Rhodes OE Jr, Shivik JA. 2003. Scavenging by vertebrates: behavioral, ecological, and evolutionary perspectives on an important energy transfer pathway in terrestrial ecosystems. Oikos. 102:225–234. 10.1034/j.1600-0706.2003.12378.x.

[arag100-B17] Fincke OM . 1992. Interspecific competition for tree holes: consequences for mating systems and coexistence in neotropical damselflies. Am Nat. 139:80–101. 10.1086/285314.

[arag100-B18] Gardner A, Smiseth PT. 2011. Evolution of parental care driven by mutual reinforcement of parental food provisioning and sibling competition. Proc R Soc Lond B Biol Sci. 278:196–203. 10.1098/rspb.2010.1171. PMC3013391 20667869

[arag100-B19] Gerstmayer PA, Monges V, Jauregui A, Colombo MA, Segura LN. 2022. Cooperative harassment strategy by the European Starling to usurp cavities and reproductive cost for the neotropical Green-barred Woodpecker. Neotrop Biodivers. 8:Article 40. 10.1080/23766808.2022.2145089.

[arag100-B20] Goldshtein A, Markman S, Leshem Y, Puchinsky M, Charter M. 2018. Nest-site interference competition with House Sparrows affects breeding success and parental care in Great Tits. J Ornithol. 159:667–673. 10.1007/s10336-018-1541-4.

[arag100-B21] Gubernick DJ . 2013. Parental care in mammals. Springer Science & Business Media.

[arag100-B22] Harrison L . 2021. Thermal ecology and plasticity in the burying beetle, *Nicrophorus orbicollis*: an observational field study [MSc thesis]. Queen's University.

[arag100-B23] Heinze G, Ploner M, Jiricka L, Steiner G. 2023. logistf: Firth's Bias-Reduced Logistic Regression. 1.26.0. 10.32614/CRAN.package.logistf .

[arag100-B24] Jacot A, Valcu M, van Oers K, Kempenaers B. 2009. Experimental nest site limitation affects reproductive strategies and parental investment in a hole-nesting passerine. Anim Behav. 77:1075–1083. 10.1016/j.anbehav.2009.01.012.

[arag100-B25] Keddy PA . 2012. Competition. 2nd ed. Springer Science & Business Media.

[arag100-B26] Keenleyside MH . 1991. Cichlid fishes: behaviour, ecology and evolution. Springer Science & Business Media.

[arag100-B27] Keller ML, Howard DR, Hall CL. 2021. The thermal ecology of burying beetles: temperature influences reproduction and daily activity in *Nicrophorus marginatus*. Ecol Entomol. 46:1266–1272. 10.1111/een.13071.

[arag100-B28] Ki B-M, Kim YM, Jeon JM, Ryu HW, Cho K-S. 2018. Characterization of odor emissions and microbial community structure during degradation of pig carcasses using the soil burial-composting method. Waste Manag. 77:30–42. 10.1016/j.wasman.2018.04.043. 30008412

[arag100-B29] Klug H, Bonsall MB. 2010. Life history and the evolution of parental care. Evolution. 64:823–835. 10.1111/j.1558-5646.2009.00854.x. 19796144

[arag100-B30] Klug H, Bonsall MB. 2014. What are the benefits of parental care? The importance of parental effects on developmental rate. Ecol Evol. 4:2330–2351. 10.1002/ece3.1083. PMC4203283 25360271

[arag100-B31] Lenth RV . 2024. 1.10.5. [emmeans: Estimated Marginal Means, aka Least-Squares Means]. CRAN. 10.32614/CRAN.package.emmeans .

[arag100-B32] Li D, Jackson RR. 2003. A predator's preference for egg-carrying prey: a novel cost of parental care. Behav Ecol Sociobiol. 55:129–136. 10.1007/s00265-003-0689-x.

[arag100-B33] Lomander A, Kätterer T, Andrén O. 1998. Carbon dioxide evolution from top- and subsoil as affected by moisture and constant and fluctuating temperature. Soil Biol Biochem. 30:2017–2022. 10.1016/S0038-0717(98)00076-5.

[arag100-B34] López-Idiáquez D, Vergara P, Fargallo JA, Martínez-Padilla J. 2018. Providing longer post-fledging periods increases offspring survival at the expense of future fecundity. PLoS One. 13:e0203152. 10.1371/journal.pone.0203152. PMC6130873 30199538

[arag100-B35] Martin K . 1997. Landscape modification and patch selection: the demography of two secondary cavity nesters colonizing clearcuts. Auk. 114:443–455. 10.2307/4089245.

[arag100-B36] Martin PR, Ghalambor CK. 2023. A case for the “competitive exclusion–tolerance rule” as a general cause of species turnover along environmental gradients. Am Nat. 202:1–17. 10.1086/724683. 37384767

[arag100-B37] Martin PR, Ghalambor CK. 2024. Is competitive ability the key adaptation to benign environments? Revisiting experiments on closely related species of tidal plants. Biol Lett. 20:20230509. 10.1098/rsbl.2023.0509. PMC11285840 38746982

[arag100-B38] Miles K . 2019. Comparative decomposition rates of piglets buried in manure: a pilot study and implications for forensic taphonomy. Can Soc Forensic Sci J. 52:174–183. 10.1080/00085030.2019.1664972.

[arag100-B39] Olsen RG, Purcell KL, Grubbs D. 2008. Nest defense behaviors of native cavity-nesting birds to European Starlings. In: Merenlender A, McCreary D, Purcell KL tech eds 2008 Proceedings of the sixth California oak symposium: today's Challenges, tomorrow's Opportunities Gen Tech Rep PSW-GTR-217. Vol. 217. US Department of Agriculture, Forest Service, Pacific Southwest Research Station. p. 457–470.

[arag100-B40] Ponce C, Alonso J. C, Argandoña G., García Fernández A, Carrasco M. 2010. Carcass removal by scavengers and search accuracy affect bird mortality estimates at power lines. Animal Conservation. 13:603–612. 10.1111/acv.2010.13.issue-6.

[arag100-B41] Potticary AL et al 2024. Revisiting the ecology and evolution of burying beetle behavior (Staphylinidae: Silphinae). Ecol Evol. 14:e70175. 10.1002/ece3.70175. PMC11336061 39170054

[arag100-B42] Pukowski E . 1933. Ökologische untersuchungen an necrophorus f. Z Morph u Ökol Tiere. 27:518–586. 10.1007/BF00403155.

[arag100-B43] Quinby BM, Belk MC, Creighton JC. 2020. Behavioral constraints on local adaptation and counter-gradient variation: implications for climate change. Ecol Evol. 10:6688–6701. 10.1002/ece3.6399. PMC7381570 32724542

[arag100-B44] R Core Team . 2024. [R: A Language and Environment for Statistical Computing].

[arag100-B45] Royle NJ, Smiseth PT, Kölliker M, editors. 2012. The evolution of parental care. 1st ed. Oxford University Press.

[arag100-B46] Schrempf SD, Burke KW, Wettlaufer JD, Martin PR. 2021. Behavioral dominance interactions between *Nicrophorus orbicollis* and *N. tomentosus* burying beetles (Coleoptera: Silphidae). PeerJ. 9:e10797. 10.7717/peerj.10797. PMC7912668 33665013

[arag100-B47] Scott MP . 1998. The ecology and behaviour of burying beetles. Annu Rev Entomol. 43:595–618. 10.1146/annurev.ento.43.1.595. 15012399

[arag100-B48] Shaffer LR, Formanowicz DR Jr. 1996. A cost of viviparity and parental care in scorpions: reduced sprint speed and behavioural compensation. Anim Behav. 51:1017–1024. 10.1006/anbe.1996.0104.

[arag100-B49] Shubeck PP . 1985. Orientation of carrion beetles to carrion buried under shallow layers of sand (Coleoptera, Silphidae). Entomol News. 96:163–166. https://ia803205.us.archive.org/16/items/biostor-77050/biostor-77050.pdf.

[arag100-B50] Shubeck PP, Blank DL. 1982. Carrion beetle attraction to buried fetal pig carrion (Coleoptera: Silphidae). Coleopterists Bull. 36:240–245. https://www.jstor.org/stable/pdf/4008059.pdf.

[arag100-B51] Suzuki S . 2000. Changing dominant–subordinate relationships during carcass preparation between burying beetle species (*Nicrophorus*: Silphidae: Coleoptera). J Ethol. 18:25–28. 10.1007/s101640070020.

[arag100-B52] Székely T, Reynolds JD. 1995. Evolutionary transitions in parental care in shorebirds. Proc Biol Sci. 262:57–64. 10.1098/rspb.1995.0176.

[arag100-B53] Teurlings IJM, Odden J, Linnell JDC, Melis C. 2020. Caching behavior of large prey by Eurasian lynx: quantifying the anti-scavenging benefits. Diversity (Basel). 12:350. 10.3390/d12090350.

[arag100-B54] Trumbo ST . 1990. Interference competition among burying beetles (Silphidae, *Nicrophorus*). Ecol Entomol. 15:347–355. 10.1111/j.1365-2311.1990.tb00816.x.

[arag100-B55] Trumbo ST . 2012. Patterns of parental care in invertebrates. In: Royle NJ, Smiseth PT, editors. The evolution of parental care. Oxford University Press. p. 81–100.

[arag100-B56] Trumbo ST . 2016a. Carcass age and reproductive costs for *Nicrophorus orbicollis* (Coleoptera: Silphidae). Environ Entomol. 45:1178–1183. 10.1093/ee/nvw114. 27550165

[arag100-B57] Trumbo ST . 2016b. Fate of mouse carcasses in a Northern Woodland. Ecol Entomol. 41:737–740. 10.1111/een.12341.

[arag100-B58] Trumbo ST . 2023. Moving the corpse to hide the evidence: horizontal as well as vertical movement is important when burying beetles cache a carcass. J Insect Behav. 36:11–19. 10.1007/s10905-023-09816-9.

[arag100-B59] Trumbo ST, Bloch PL. 2000. Habitat fragmentation and burying beetle abundance and success. J Insect Conserv. 4:245–252. 10.1023/A:1011390215088.

[arag100-B60] Trumbo ST, Bloch PL. 2002. Competition between *Nicrophorus orbicollis* and *N. defodiens*: resource locating efficiency and temporal partitioning. Northeast Nat (Steuben). 9:13–26. 10.1656/1092-6194(2002)009[0013:CBNOAN]2.0.CO;2.

[arag100-B61] Trumbo ST, Philbrick PKB, Stökl J, Steiger S. 2021. Burying beetle parents adaptively manipulate information broadcast from a microbial community. Am Nat. 197:366–378. 10.1086/712602. 33625971

[arag100-B62] Trumbo ST, Sikes DS. 2021. Resource concealment and the evolution of parental care in burying beetles. J Zool. 315:175–182. 10.1111/jzo.12916.

[arag100-B63] Turner KL, Abernethy EF, Conner LM, Rhodes OE Jr, Beasley JC. 2017. Abiotic and biotic factors modulate carrion fate and vertebrate scavenging communities. Ecology. 98:2413–2424. 10.1002/ecy.1930. 28628191

[arag100-B64] Vangenne YD, Sheppard B, Martin PR. 2023. Behavioral dominance interactions between two species of burying beetles (*Nicrophorus orbicollis* and *Nicrophorus pustulatus*). PeerJ. 11:e16090. 10.7717/peerj.16090. PMC10676716 38025751

[arag100-B65] Verheggen F et al 2017. The odor of death: an overview of current knowledge on characterization and applications. BioScience. 67:600–613. 10.1093/biosci/bix046.

[arag100-B66] Wettlaufer JD, Burke KW, Beresford DV, Martin PR. 2021. Partitioning resources through the seasons: abundance and phenology of carrion beetles (Silphidae) in southeastern Ontario, Canada. Can J Zool. 99:961–973. 10.1139/cjz-2021-0081.

[arag100-B67] Wettlaufer JD, Ye A, MacMillan HA, Martin PR. 2023. A test of the competitive ability–cold tolerance trade-off hypothesis in seasonally breeding beetles. Ecol Entomol. 48:55–68. 10.1111/een.13201.

