# Supplementary material for: Why do burying beetles bury? Competition and the fitness benefits of parental care
Source: Behav Ecol. 2026 Aug 29;37(6):arag100. doi: 10.1093/beheco/arag100 (PMC13641174; doi:10.1093/beheco/arag100)
Supplement: Supplementary file 1 [file arag100_supplementary_data.docx]

Supplemental Material for:

Why do burying beetles bury? Competition and the evolution of parental care

Behavioral Ecology

Erin Bolger^*^ and Paul R. Martin

*Department of Biology, Queen’s University, Kingston, Ontario, Canada K7L 3N6*

* Correspondence: [bolgerin@gmail.com](mailto:bolgerin@gmail.com)

**Supplemental Figures**

FIGURE S1 Experimental set-up to test the fitness benefits of burial. Each site had two buried tubes (burial and non-burial treatments) (**a**, side view of the PVC pipe breeding apparatus used for our experiments). We attached a flowerpot with its bottom removed to each buried tube using duct tape (**b**, side view of a buried tube with flowerpot attached), which prevented the beetles from moving the mouse out of the PVC pipe and burying elsewhere. We then placed a thawed mouse carcass on top of each buried tube, within each flowerpot, and provided ramps made of rocks, logs, or bark (shown) to allow invertebrates to access the carcass (**c**, top view of a tube and flowerpot with a mouse carcass and bark ramp). After burying beetles had commenced breeding behaviors, we removed the flowerpot from the site so that sites were as natural as possible (**d**, top view of a tube with flowerpot removed, with an arrow indicating the edge of the buried tube).

**Supplemental Methods**

Nicrophorus *burial*

Both the male and the female participate in burying and creating a brood chamber. The depth of burial depends on the soil type where burying occurs, ambient temperature, the species and size of beetle burying the carcass, and the size of the carcass, with some species burying carcasses under leaf litter or vegetation, and other species burying deeper into soil (Potticary et al. 2024). The prevalence of burial as a strategy throughout *Nicrophorus* suggests that burial is an adaptation for reproduction.

*Experimental methods*

Mouse carcasses were either donated by the Queen’s University Health Sciences breeding facilities or purchased from a retailer (Canadian Mouseman, Medicine Hat, Alberta; https://canadianmouseman.com/). Mice were culled using carbon dioxide gas and immediately frozen at -20C. We thawed mice for 48 hours prior to experimental use.

In 2023, we added rocks and logs or large pieces of bark beside flowerpots to allow the beetles (and other invertebrates) to access the carcass (though they could fly in as well) (Figure S1c); beetles were attracted to carcasses naturally, forming breeding pairs after arrival at sites. In 2024, we first collected *N. orbicollis* using nonlethal pitfall traps baited with mice (thawed from frozen for 48 hours). The beetles could have initiated reproduction in the pitfall traps, so we allowed them five days to recover in containers in the laboratory prior to using them in the experiment. In the laboratory, we sexed each beetle, fed each beetle a small piece of ground beef, and provided them with a damp paper towel. Four days after feeding, we set out a pair of beetles along with the mouse carcass. To ensure the beetles would not fly away from the carcass when we initially placed them at the site, we covered the top of the flowerpot with a piece of paper attached with masking tape for 24 hours (2024 only).

Temperature: To calculate average temperature, we downloaded daily historical climate data from a nearby weather station (Hartington station, 33 km from QUBS) for 2023 and 2024 (Environment and Climate Change Canada 2025), found the average daily temperature for each day that an experiment was ongoing, and averaged those values over the ten-day experimental window to find the average temperature for each experiment at each site.

*Statistical Methods*

We repeated analyses for each year separately to investigate whether a different modeling approach influenced the main results.

We standardized average temperature values by centering and scaling (i.e., subtracting the mean and dividing by 1 standard deviation) using the function ‘scale’ from the base R package (R Core Team 2024), and used the standardized values as a predictor in our models (Bates et al. 2015). The resulting model predictors were treatment*year + treatment*z.avg.temp.

We assessed the fit and assumptions of binomial generalized linear mixed-effects models (glmer) and binomial generalized linear models (glm) by simulating scaled residuals from the fitted models (with the R package DHARMa; version 0.4.7; Hartig, 2024), generating QQ plots (to detect deviations from a binomial distribution) and plots of residuals against model predicted values (to highlight outliers), and testing simulated residuals for zero inflation.

Most glmer models had issues when estimating variance due to the lack of variation in the non-burial treatment (no successes). To accommodate these issues, we used Firth’s Bias-Reduced Logistic Regression (function ‘logistf’ from the R package logistf, version 1.26.0; Heinze et al. 2023). We did not include site as a random effect in this model because Firth’s Correction is not compatible with mixed-effects models, and all non-burial treatments had the same value in some cases (e.g., success vs failure), regardless of location. We checked the fit of logistf models using the function “heatmap.fit” from the R package heatmapFit (version 2.0.4; Esarey et al. 2016), because the packages logistf and DHARMa were not compatible.

We plotted results in R using the R package ggplot2 (version 4.0.1; Wickham 2016). We generated 95% confidence intervals for plotting using bootstrapping, which resamples data and calculates the means of each resample, and then calculates the mean and 95% confidence limits of those mean estimates (functions ‘boot’ and ‘boot.ci’ from the R package boot (version 1.3-31; Davison and Hinkley 1997; Canty and Ripley 2024); N = 1,000 resamples). We used the raw data rather than the model predicted values for plots and 95% confidence interval estimates to avoid issues arising from the lack of variation in the reproductive success results (no reproductive success in the non-burial treatment).

**Supplemental Results**

Models run separately for each year yielded similar results to the main analysis. The non-burial treatment had lower reproductive success than the burial treatment in both 2023 (Firth's Bias-Reduced Logistic Regression (logistf), coef = -4.44, SE = 1.47, *χ^2^* = 28.1, *p* < 0.001) and 2024 (logistf, coef = -3.53, SE = 1.55, *χ^2^* = 10.1, *p* = 0.001). Failure due to vertebrates varied across years: we found weak evidence for higher vertebrate scavenging in the non-burial treatment in 2023 (logistf, coef = 0.53, SE = 0.68, *χ^2^* = 0.63, *p* = 0.43), and strong evidence in 2024 (logistf, coef = 2.07, SE = 0.91, *χ^2^* = 5.93, *p* = 0.015). Failure due to invertebrates also varied across years: we found strong evidence for higher invertebrate scavenging in the non-burial treatment in 2023 (logistf, coef = 2.65, SE = 0.65, *χ^2^* = 20.74, *p* < 0.001); and weak evidence in 2024 (logistf, coef = 0.80, SE = 0.91, *χ^2^* = 0.79, *p* = 0.37).

**Supplemental Discussion**

**Temperature**

Temperature may play a large role in mediating the relationship between vertebrate and invertebrate pressure (DeVault et al. 2004). Higher temperatures increase the activity of invertebrates and microbial competitors, which speeds their discovery of carcasses and hastens the consumption of carrion, rendering carcasses unusable for vertebrates more quickly; vertebrates tend to have lower scavenging efficiency with higher temperatures (DeVault et al. 2004). Vertebrates are also more likely to scavenge older carcasses during the winter, when lower temperatures limit microbial and invertebrate activity, slowing decay (DeVault et al., 2003; DeVault et al., 2004). The temperature range during our experiment was relatively narrow (the lowest ten-day average was 18.6°C and the highest was 22.4°C), which likely prevented us from finding any effect of temperature on invertebrate versus vertebrate scavenging activity; differences in relative rates of scavenging are most often seen at more extreme temperatures (DeVault et al. 2004).

**Impact of microclimate**

Unburied carcasses remained exposed to the environment, sometimes leading to the desiccation of those carcasses. Desiccation occurs when invertebrates and microbes on the carcass and in the surrounding soil are not able to fully digest a carcass, frequently when weather conditions are warm and dry (Payne 1965). Burying beetles have lower reproductive success on drier carcasses and avoid desiccated carrion for feeding and breeding (Hayes et al. 2025). Burial of carcasses likely reduces desiccation by maintaining moister, cooler, and more stable temperature conditions underground (Pokines and Symes 2013; Harrison 2021). Unburied carcasses in our experiment were impacted by desiccation much more frequently than buried carcasses; however, desiccation never caused the failure of an unburied carcass on its own – it was observed exclusively in conjunction with invertebrate competition, which hastened desiccation by increasing exposed carcass surface area and reducing carcass mass. Thus, burial likely serves a dual purpose in reducing the invertebrate competitor activity that hastens desiccation, as well as reducing desiccation by buffering microclimate.

**Additional benefits of burying**

Carcass burial likely confers additional fitness benefits to burying beetles that we could not detect in our experiment because of the complete failure of non-burial treatments due to invertebrate and vertebrate competition. Other fitness benefits may include reduced microbial competition, reduced competition with other *Nicrophorus*, improved conditions for egg and larval development, and provision of a physical brood chamber. Burying beetles often avoid breeding on carcasses in the later stages of decomposition (i.e., microbial consumption), (Putman 1983; Trumbo and Newton 2022), likely because carcasses diminish in nutritional value as they decompose (Trumbo 2016a). Burial may mitigate decomposition due to cooler temperatures underground, reducing microbial activity and slowing decomposition (Lomander et al. 1998; Pokines and Symes 2013; Harrison 2021), and in our experiment, buried carcasses never completely decomposed and only unburied carcasses showed signs of major decomposition. Burying beetles also compete for carcasses with other *Nicrophorus* species or members of their own species (Potticary et al. 2024). Burying beetles can usurp carcasses from subordinate competitors, even when carcasses are buried (Trumbo 1990; Scott 1994); however, burial (in combination with carcass preparation and maintenance) likely reduces competition with other *Nicrophorus* species by reducing their ability to locate and take over carcasses (Trumbo 1994; Trumbo et al. 2021; Trumbo and Sikes 2021). Burial may also provide favourable conditions for rearing offspring: *Nicrophorus orbicollis* adjusts its brood sizes depending on temperature, and typically produces smaller broods with lower offspring mass at warmer temperatures (Quinby et al. 2020), egg mortality is higher at warmer temperatures, and larval mass is lower when temperatures are warmer or more variable (Nisimura et al. 2002; Harrison 2021; Keller et al. 2021). Underground burial provides cooler and more stable temperatures, which likely increases fitness through improved offspring quality. During carcass burial, *N. orbicollis* usually creates an underground breeding chamber that facilitates parental attendance of offspring, which could increase offspring fitness through increased food provisioning and offspring defense (Scott and Traniello 1990; Royle et al. 2012; Smiseth et al. 2014). However, burial and a breeding chamber is not essential for breeding; *N. orbicollis* can achieve high levels of reproductive success without either (lab experiments demonstrate this) (Trumbo 1992; Benowitz and Moore 2016). Though the benefits described above were not explicitly tested in our study and none of these factors caused failure in our experiments, these factors could provide secondary fitness benefits in addition to reducing invertebrate and vertebrate competition.

**Supplemental References (not included in the main text)**

Bates D, Mächler M, Bolker B, Walker S. 2015. Fitting Linear Mixed-Effects Models Using lme4. Journal of Statistical Software. 67(1):1–48. doi:10.18637/jss.v067.i01.

Benowitz KM, Moore AJ. 2016. Biparental care is predominant and beneficial to parents in the burying beetle *Nicrophorus orbicollis* (Coleoptera: Silphidae). Biological Journal of the Linnean Society. 119(4):1082–1088. doi:10.1111/bij.12830.

Canty A, Ripley B. 2024. boot: Bootstrap R (S-Plus) Functions.

Davison AC, Hinkley DV. 1997. Bootstrap methods and their applications. Cambridge: Cambridge University Press.

Esarey J, Pierce A, Du J. 2016. heatmapFit: Fit Statistic for Binary Dependent Variable Models.

Hartig, F. 2024. DHARMa: Residual Diagnostics for Hierarchical (Multi-Level / Mixed) Regression Models.

Hayes TE, Lassérès L, Yang LH. 2025. Moisture and competition constrain ephemeral resource quality for burying beetle reproduction. Ecology. 106(6):e70127. doi:10.1002/ecy.70127.

Nisimura T, Kon M, Numata H. 2002. Bimodal life cycle of the burying beetle *Nicrophorus quadripunctatus* in relation to its summer reproductive diapause. Ecological Entomology. 27(2):220–228. doi:10.1046/j.1365-2311.2002.00400.x.

Payne JA. 1965. A summer carrion study of the baby pig *Sus scrofa linnaeus*. Ecology. 46(5):592–602. doi:10.2307/1934999.

Pokines J, Symes SA, editors. 2013. Manual of forensic taphonomy. Boca Raton: CRC Press.

Ponce C, Alonso JC, Argandoña G, García Fernández A, Carrasco M. 2010. Carcass removal by scavengers and search accuracy affect bird mortality estimates at power lines. Animal Conservation. 13(6):603–612. doi:10.1111/j.1469-1795.2010.00387.x.

Putman R. 1983. Carrion and dung : the decomposition of animal wastes. London : E. Arnold.

R Core Team. 2024. R: A Language and Environment for Statistical Computing.

Scott MP. 1994. Competition with flies promotes communal breeding in the burying beetle, *Nicrophorus tomentosus*. Behavioral Ecology and Sociobiology. 34(5):367–373. doi:10.1007/BF00197007.

Scott MP, Traniello JFA. 1990. Behavioural and ecological correlates of male and female parental care and reproductive success in burying beetles (*Nicrophorus* spp.). Animal Behaviour. 39(2):274–283. doi:10.1016/S0003-3472(05)80871-1.

Smiseth PT, Andrews CP, Mattey SN, Mooney R. 2014. Phenotypic variation in resource acquisition influences trade-off between number and mass of offspring in a burying beetle. Journal of Zoology. 293(2):80–83. doi:10.1111/jzo.12115.

Trumbo ST. 1992. Monogamy to communal breeding: exploitation of a broad resource base by burying beetles (*Nicrophorus*). Ecological Entomology. 17(3):289–298. doi:10.1111/j.1365-2311.1992.tb01060.x.

Trumbo ST. 1994. Interspecific competition, brood parasitism, and the evolution of biparental cooperation in burying beetles. Oikos. 69(2):241–249. doi:10.2307/3546144.

Trumbo ST, Newton AF. 2022. Microbial volatiles and succession of beetles on small carrion. Ecological Entomology. 47(5):758–769. doi:10.1111/een.13160.

Wickham H. 2016. ggplot2: Elegant Graphics for Data Analysis.
